# Effect of the Initial Deformity in the Coronal Plane on Postoperative Outcome of Proximal Humeral Fractures

**DOI:** 10.1111/os.13690

**Published:** 2023-05-17

**Authors:** Fei Dai, Ming Xiang, Qing Zhang, Jinsong Yang, Yiping Li

**Affiliations:** ^1^ Sichuan Provincial Orthopaedics Hospital Chengdu China; ^2^ Upper Limb Department Sichuan Provincial Orthopaedic Hospital Chengdu China; ^3^ Tenth People's Hospital Shanghai China

**Keywords:** Complications, Initial deformity, Maintenance of reduction, Proximal humeral fractures

## Abstract

**Objective:**

There is currently no consensus on proximal humerus fractures with an initial deformity in the coronal plane who are better off with plates or nails, so we designed this study. To compare the effect of the initial deformity in the coronal plane of proximal humerus fractures on postoperative outcomes, we compare the maintenance of reduction in procedures utilizing plates and nails, and analyzed the subsequent occurrence of complications to explore whether the initial deformity should dictate the fixation approach.

**Methods:**

The clinical data of patients with proximal humerus fractures who were hospitalized and underwent surgical treatment in our hospital from January 2016 to December 2020 were reviewed. Postoperative functional scores (American Shoulder and Elbow Surgeons, ASES; Constant‐Murley Score, CMS), Neck‐shaft angle (NSA), Quality of fracture reduction, Deltoid Tuberosity Index (DTI), and complications were compared among cases with initial varus, normal, or valgus deformities.

**Results:**

We included 131 patients, 56 males and 75 females, with a mean age of 60.89 ± 5.53 years (range 50–76) and a mean follow‐up duration of 16.63 ± 6.78 months (range 12–48). Of these, 29 cases had initial varus displacement, 71 had a normal NSA, and 31 had initial valgus displacement. Seventy‐five were treated with a locking plate and 56 with a nail. After open reduction and internal fixation, the NSA was restored to normal (−135°) in all patients in all groups (*P* > 0.05). There was a significant difference in NSA changes at the last follow‐up; 2.93° ± 2.12° in the varus group, 1.77° ± 1.18° in the normal group, and 2.32° ± 1.64° in the valgus group, with the highest change occurring in the varus group. There was no significant difference in the range of motion or functional scores including ASES and CMS among the three groups (*P* > 0.05). The complication rate of 20.7% in the varus group was significantly higher than the complication rates of 12.7% in the normal and 12.9% in the valgus groups (*P* < 0.05).

**Conclusions:**

While proximal humerus fractures with initial coronal displacement (varus, normal, and valgus) show similar postoperative functional outcomes, varus fractures have a higher rate of complications. The nail provides better maintenance of reduction than the locking plate, especially in varus fractures.

## Graphical abstract

While proximal humerus fractures with initial coronal displacement (varus, normal, and valgus) have similar postoperative function, varus fractures have a higher complication rate. The nail provides better maintenance of reduction relative to the locking plate, especially in varus fractures.

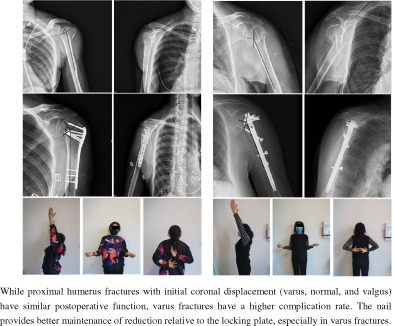

## Introduction

Current epidemiological studies suggest that proximal humerus fractures are very common, representing 4%–10% of all fractures.^1^ This proportion is increased when surgical intervention cases are examined, as approximately 30% of proximal humerus fractures are treated surgically.^2^ As the population ages, the incidence of proximal humerus may continue to rise and the need for surgical treatment may gradually increase. As such, appropriate treatment strategies are essential to reduce the risk of poor prognosis and loss of self‐care ability in these populations. The use of the locking plate technique for treating proximal humerus fractures has gained traction, but postoperative adverse outcomes and complications remain common. These include varus displacement, screw cut‐out, and avascular necrosis of the humeral head.^3^ Intramedullary nailing, compared with eccentric fixation of plates, has the intrinsic advantage of resisting varus and rotational stresses and is theoretically more favorable for maintaining fracture reduction, especially when varus deformity is present. However, this technique is still associated with some complications such as iatrogenic rotator cuff injury and nonunion of the fracture.^4^, ^5^, ^6^ There are many studies about proximal humerus plate or nail treatment, but few have tested which internal fixation method is better for varus or valgus fractures. Therefore we lack consensus on the optimal treatment of proximal humerus fractures.

The treatment of proximal humeral fracture requires consideration of patient characteristics, fracture types, and surgeon preferences. An increasing number of studies have focused on the initial varus deformity in proximal humerus fractures, arguing that this pathological feature, particularly medial cortical comminution, is more likely to cause the failure of internal fixation, worse function, and higher rates of surgical revision. Current methods for managing varus displacement of the humeral head include the use of calcar screws, allograft fibula implantation, dual plate fixation,^7^ etc., but these can lead to adverse immune responses, infections, and joint stiffness. At the same time, there is considerable variation in the definition of varus deformity across relevant studies. In short, how the initial deformity in the coronal plane of a proximal humerus fracture affects postoperative outcomes has not been fully investigated.^8^, ^9^, ^10^, ^11^ Therefore, we retrospectively analyzed the clinical data of patients with proximal humerus fractures who underwent surgical treatment at our institution.

The objectives of the study were to: (i) analyze the influence of the initial proximal humerus fracture deformity in the coronal plane on postoperative outcomes; (ii) compare the maintenance of the reduction with plates and nails; and (iii) analyze the incidence of postoperative complications.

## Materials and Methods

Study inclusion criteria were: (i) age ≥ 50 years; (ii) Part 2, 3 and 4 fractures of Neer classification; (iii) treated with Philos locking plate or Multiloc intramedullary nail (DePuy Synthes, Westchester, PA, USA); and (iv) complete follow‐up data were available and the follow‐up was longer than 1 year. We excluded: (i) isolated greater tuberosity fractures or lesser tuberosity fractures; (ii) history of previous surgery on the affected shoulder; (iii) open fractures; (iv) pathological fracture (e.g. cancer or osteoporosis‐related); and (v) combined massive irreparable rotator cuff tears.

This study was approved by the Sichuan orthopedic hospital Institutional Review Board (20210134). We included 131 cases of proximal humerus fractures who were treated with a locking plate or intramedullary nail fixation from January 2016 to December 2020 in our hospital and who met our inclusion criteria. The fractures are divided into 2, 3, and 4 parts according to the Neer classification. X‐rays of the shoulders were taken preoperatively, postoperatively, and at the final follow‐up. CT or MRI were performed as indicated.

### Surgical Approach

All patients underwent surgery under general anesthesia with local brachial plexus anesthesia. The beach chair position was used, and the posterior aspect of the shoulder joint on the operated side was padded high to ensure adequate passive flexion and extension of the affected limb during surgery.

#### Philos Locking Plate Group

A standard deltopectoral approach or delta‐split approach was utilized depending on the fracture type and physician preference. After exposing the fracture, the rotator cuff attached at the greater and lesser tuberosity was sutured with 5# Ethibond sutures for subsequent reduction traction. Two to three Kirschner wires were used to temporarily secure a proximal humeral fracture fragment to aid reduction and correct rotation and varus/valgus positioning of the humeral head. After reduction, the fracture was fixed using a Philos locking plate (DePuy Synthes, Westchester, PA, USA). The plates were placed approximately 5 to 8 mm from the highest point of the greater tuberosity and 2 to 4 mm posterior to the intertubercular groove. Intraoperative fluoroscopic shoulder anteroposterior and lateral and axillary images were then obtained to ensure correct fracture reduction, plate position, and screw length. For patients with concomitant rotator cuff tears, anchors were placed to repair the rotator cuff.

#### Multiloc Intramedullary Nail Group

The split deltoid approach was used. The proximal humerus and the rotator cuff were exposed, and the rotator cuff with the greater and lesser tuberosity attached was sutured with 5# Ethibond sutures to facilitate intraoperative control of the humeral head and reduction of the greater and lesser tuberosity fracture fragments. The fracture was reduced by using a Kirschner wire joystick technique. At 6 to 8 mm posterior to the biceps tendon, which is the highest point of the humeral head, the rotator cuff was incised and a guide wire was placed. After fluoroscopic confirmation of the correct entry point and guide wire orientation, the access hole was expanded, and based on the medullary cavity diameter, a Multiloc intramedullary nail (DePuy Synthes, Westchester, PA, USA) was inserted. Fracture reduction and screw length were then confirmed by fluoroscopy after screw placement. For patients with rotator cuff tears, repair of the rotator cuff was indicated.

### Postoperative Management

Active elbow and wrist movements and painless passive training of the shoulder were allowed for 1 to 2 days postoperatively. Passive activity started 1–2 weeks after the operation. Active functional exercise was started at 4 weeks. Shoulder strength exercises generally commenced 3 months after surgery.

### Outcome Measures

All patients were followed up regularly postoperatively, and radiographs of the shoulders were obtained and assessed using relevant review systems.

**Fig. 1:**
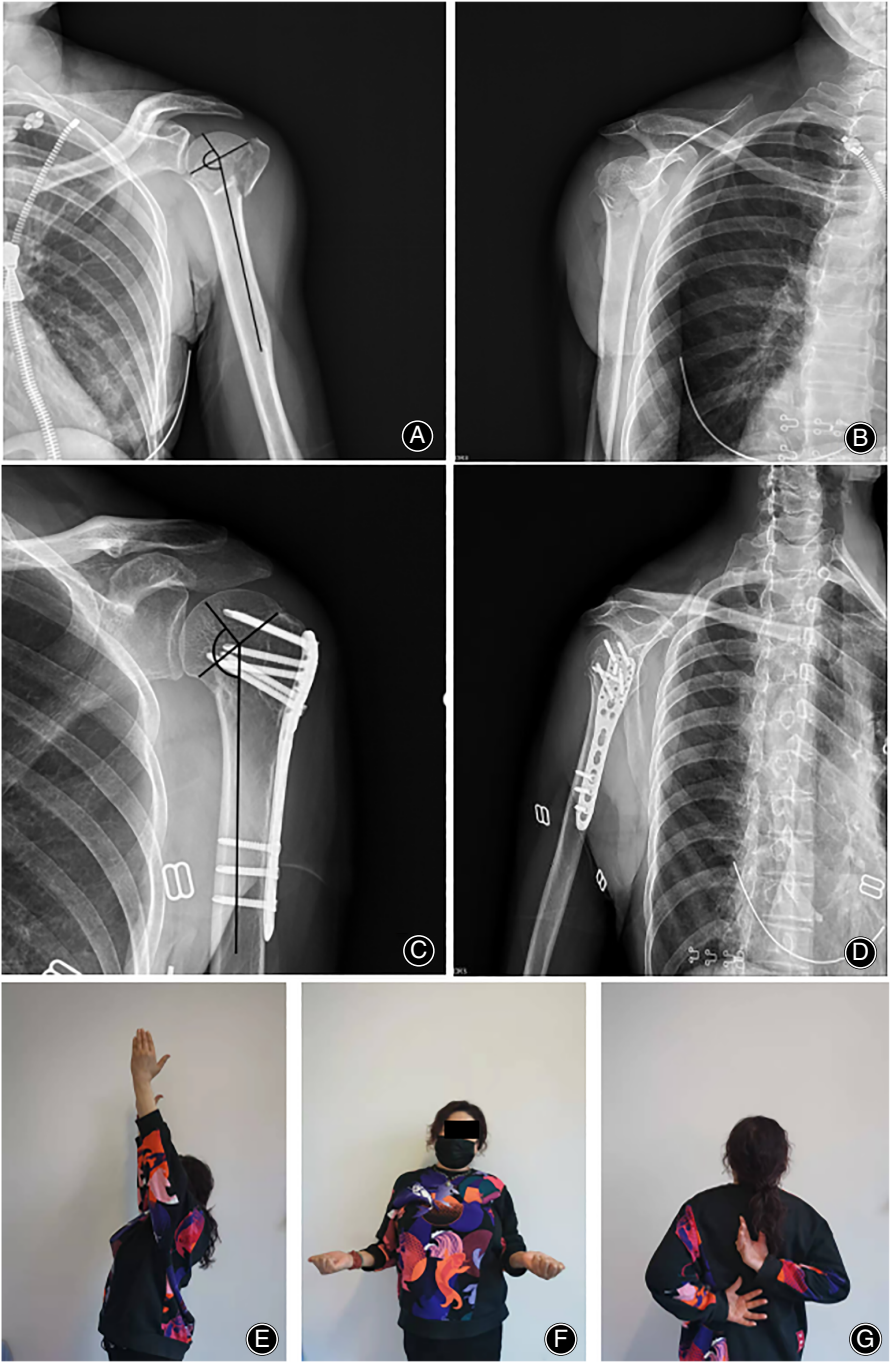
53 year old female, valgus fracture with an initial NSA of 157°(A, B); Treated with plate fixation with an NSA of 136°at last follow‐up (C, D); Shoulder function is excellent at last follow‐up (E–G)

**Fig. 2:**
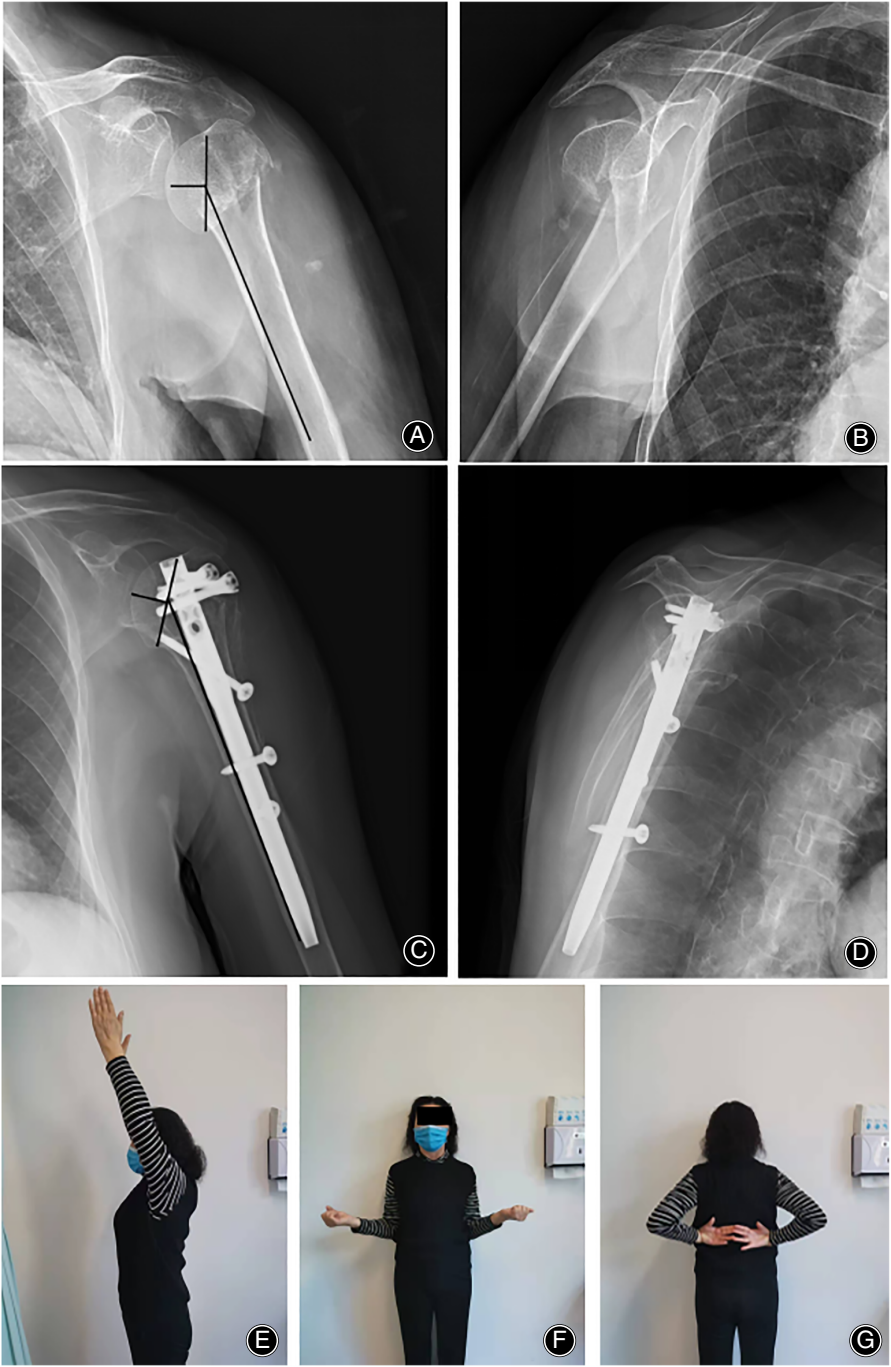
62 year old female, varus fracture with an initial NSA of 112°(A, B); Treated with nails fixation with an NSA of 131° at last follow‐up (C, D); Shoulder function is satisfactory at last follow‐up (E–G)

The humeral neck‐shaft angle (NSA) was measured based on the method of Agudelo *et al*.^12^ The normal NSA is considered to be 130° with a range of 120°–140°; we defined varus as an angle of <120° and valgus as >140°. The NSA was measured preoperatively, postoperatively, and at the last follow‐up. Typical cases are shown in Figs 1 and 2.

Pain was assessed by using a visual analog scale (VAS). This scale range from 0 to 10 points, with 10 indicating intolerable pain and 0 indicating a pain‐free state.

Shoulder function was evaluated using the American Shoulder and Elbow Surgeons (ASES) score. A full scale of 100 is assigned to this score, including subjective pain (50%) and patient assessment of viability (50%). Higher scores indicate better shoulder function.

Shoulder function was assessed using the Constant‐Murley score (CMS). The score includes the patient's pain condition (15 points), the standard of daily living (20 points), muscle strength (25 points), and shoulder range of motion (40 points) on a 100‐point scale; a higher score indicates better shoulder function.

The quality of the fracture reduction was assessed according to Bai *et al*.^13^ loss of reduction was defined as varus malunion with a neck shaft angle decrease of >10°.

**Fig. 3:**
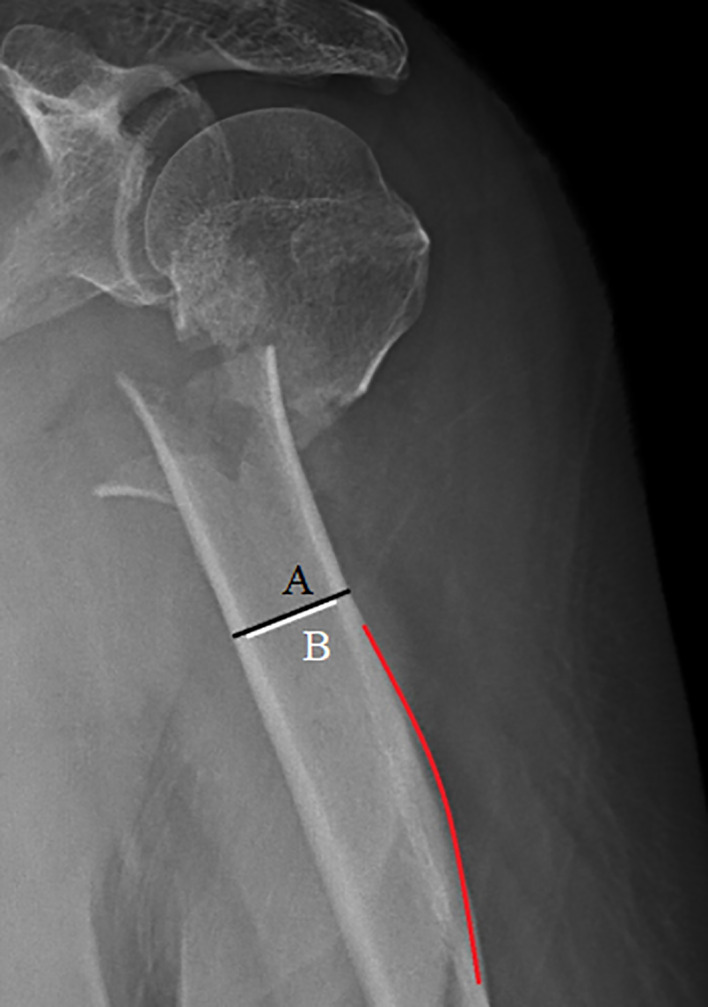
The deltoid tuberosity index is measured directly proximal to the deltoid tuberosity (red line), where the outer cortical borders become parallel. Total humeral width (A, black) and medullary cavity width (B, white) are measured, and DTA = A/B

The deltoid tuberosity index^14^ (DTI) was measured directly at a position where the cortical bone layers are parallel to each other above the deltoid tuberosity. Total humeral width (A) and medullary cavity width (B) were measured in all cases. Index values were then obtained using the formula DTI = A/B. A DTI ≥1.4 indicates a better bone condition and a DTI <1.4 indicates osteoporosis. Larger values of DTI indicate a better osseous condition. (Fig. 3).

Complications assessments included avascular necrosis, screw cut‐out, infection, and tuberosity malunion or resorption. Avascular necrosis (AVN) of the humeral head was assessed by using plain X‐ray imaging or MRI. We suspected early osteonecrosis in the presence of local cystic and/or sclerotic changes in the humeral head were seen on X‐ray; this indicated the need for further MRI to confirm the diagnosis. Later‐stage osteonecrosis was determined from radiographs in the presence of flattening, collapse, and degenerative changes of the humeral head.^15^ Screw cut‐out was defined as screw penetration into the glenohumeral joint on subsequent radiographic imaging. Tuberosity malunion or resorption was defined as tuberosities that have undergone healing but were displaced >5 mm; tuberosity absence on imaging was termed tuberosity resorption.

### Statistical Analyses

Data were analyzed by using SPSS 22.0 (IBM, Armonk, NY). Count data are expressed as counts or percentages, while continuous data are described by mean ± standard deviation. Patients were divided into three groups based on the clinical presentation: initial varus, initial valgus, and the normal group. Between‐group comparisons were made by using *k*‐independent samples nonparametric tests. Patients were divided into plate and nail groups based on the surgical intervention; between‐group comparisons were performed using chi‐square tests and independent samples t‐tests (Fisher exact testing was used if the chi‐square test is not met). The significance level threshold was set at α = 0.05 for all tests.

## Results

### General Cohort Data

We included 131 patients, including 56 males and 75 females, with a mean age of 60.89 ± 5.53 years (range 50–76) and a mean follow‐up time of 16.63 ± 6.78 months (range 12–48). Of these, 29 cases had an initial varus displacement, 71 had a normal neck shaft angle, and 31 had an initial valgus displacement. The three groups were similar at baseline by age, sex, side, length of follow‐up, Neer classification, DTI, and mode of internal fixation (Table 1). Seventy‐five patients were treated with a locking plate and 56 with a nail; 23 patients underwent intraoperative repair of the rotator cuff, including 12 in the plate group and 11 in the nail group.

**TABLE 1:** General information of the three groups (mean ± SD or cases [%])

	Varus (n = 29)	Normal (n = 71)	Valgus (n = 31)	*p*‐value
Age (years)	57.03 ± 10.54	57.44 ± 9.69	60.13 ± 7.95	0.569
Gender				0.433
Male	15 (51.7)	27 (38)	14 (45.2)	
Female	14 (48.3)	44 (62)	17 (54.8)	
Side				0.433
Left	13 (44.8)	38 (53.5)	13 (41.9)	
Right	16 (55.2)	33 (46.5)	18 (58.1)	
Follow‐up (months)	15.9 ± 5.19	16.51 ± 6.39	17.61 ± 8.8	0.759
DTI	1.49 ± 0.21	1.53 ± 0.18	1.50 ± 0.17	0.354
Neer classification				0.656
2 part	8 (27.6)	23 (32.4)	12 (38.7)	
3 part	14 (48.3)	25 (35.2)	12 (38.7)	
4 part	7 (24.1)	23 (32.4)	7 (22.6)	
Mode of internal fixation				0.143
Locking plate	18 (62.1)	44 (62)	13 (41.9)	
Intramedullary nails	11 (37.9)	27 (38)	18 (58.1)	

Abbreviation: DTI, Deltoid tuberosity index.

### Reduction and Functional Outcomes

The initial NSA was 111.79° ± 4.23° in the varus group, 129.56° ± 5.72° in the normal group, and 145.42° ± 4.01° in the valgus group (*P* < 0.05). After open reduction and internal fixation, the NSA was restored to normal (approximately 135°) in all patients; this did not differ by group (*P* > 0.05). However, at the last follow‐up, there was a statistically significant difference in NSA changes with 2.93° ± 2.12° in the varus group which saw the largest change, and 1.77° ± 1.18° in the normal group, and 2.32° ± 1.64° in the valgus group. There were no cases of loss of reduction at the last follow‐up.

At the last follow‐up, anterior flexion was 133.10° ± 17.89° in the varus cohort, 135.21° ±17.6° in the normal group, and 137.41° ± 14.82° in the valgus group, without significant differences between groups (*P* > 0.05). External rotation was 32.06° ± 8.29°, 31.40° ± 7.32°, and 32.90° ±8.14°, respectively (*P* > 0.05). The ASES score was 79.06 ± 9.96 in the varus group, 79.35 ± 10.52 in the normal group, and 80.45 ± 9.31 in the valgus group (*P* = 0.285). The CMS was 79.00 ± 10.18, 79.08 ± 10.78, and 80.58 ± 9.57, respectively (*P* > 0.05) (Table 2).

**TABLE 2:** Comparison of reduction and function among three groups (mean ± SD or cases [%])

	Varus (n = 29)	Normal (n = 71)	Valgus (n = 31)	*p*‐value
Initial NSA	111.79 ± 4.23	129.56 ± 5.72	145.42 ± 4.01	0.000^a^
Postoperative NSA	134.79 ± 1.71	135.02 ± 1.54	135.74 ± 1.59	0.06
Last follow‐up NSA	131.86 ± 3.17	133.26 ± 2.03	133.54 ± 2.37	0.106
NSA changes	2.93 ± 2.12	1.77 ± 1.18	2.32 ± 1.64	0.008^a^
Anterior flexion	133.10 ± 17.89	135.21 ± 17.67	137.41 ± 14.82	0.734
External rotation	32.06 ± 8.29	31.40 ± 7.32	32.90 ± 8.14	0.616
VAS	0.82 ± 1.03	0.80 ± 1.77	0.54 ± 0.92	0.525
ASES	79.06 ± 9.96	79.35 ± 10.52	80.45 ± 9.31	0.849
CM	79.00 ± 10.18	79.08 ± 10.78	80.58 ± 9.57	0.808
Complication				0.000^a^
Yes	6(20.7)	9(12.7)	4(12.9)	
No	23(79.3)	62(87.3)	27(87.1)	

Abbreviations: ASES, American Shoulder and Elbow Surgeons score; CM, Constant‐Murley score; NSA, neck shaft angle; VAS, visual analog scale.

^a^ Indicates that there is a significant difference among three groups.

### Surgical Complications

Among the 131 patients, the rate of complications was 14.5% (19/131), including six cases in the varus group (20.7%, 6/29), nine cases in the normal group (12.7%, 9/71), and four cases in the valgus group (12.9%, 4/31). The varus group has a significantly higher complication rate than the normal and valgus groups (*P* < 0.05). In the varus group, three patients had a screw cut‐out, one patient had an AVN combined with screw cut‐out, one patient had a tuberosity malunion, and one patient had an infection. In the normal group, two patients had a screw cut‐out, one AVN, four tuberosity malunions, one tuberosity resorption, and one infection. In the valgus group, tuberosity malunion was found in two patients and tuberosity resorption in two patients. The screws were surgically removed in patients with screw perforations and AVN, the patients have pain relief and can live a self‐care life without further surgery. Two infected patients underwent surgical removal of the internal fixations, debridement, and infection control.

### NSA Changes

Preoperatively and postoperatively, the NSA was similar in the two groups; but for the NSA changes, the plate group was significantly larger than the nail group (2.44° ± 1.89° *vs* 1.78° ± 0.98°, *P* = 0.02). In the varus group, the change in NSA was significantly larger in the plate group than in the nail group (3.66° ± 2.19 *vs* 1.72° ± 1.34, *P* = 0.007). In both the normal and valgus groups, NSA changes were similar in the plate and nail groups (normal: 1.84° ± 1.31° *vs* 1.66° ± 0.96, *P* = 0.522; valgus: 2.76 ± 2.35°*vs* 2.0 ± 0.76, *P* = 0.275) (Table 3).

**TABLE 3:** Comparison of NSA changes among three groups (mean ± SD)

NSA	Plate(n = 75)	Nails(n = 56)	t‐value	*p*‐value
Mean	2.44 ± 1.89	1.78 ± 0.98	2.353	0.02^a^
Varus	3.66 ± 2.19	1.72 ± 1.34	2.631	0.007^a^
Normal	1.84 ± 1.31	1.66 ± 0.96	0.598	0.522
Valgus	2.76 ± 2.35	2.0 ± 0.76	1.303	0.275

^a^ Indicates that there is a significant difference.

## Discussion

We classified the initial deformity in the coronal plane as varus, normal, or valgus based on the neck shaft angle measured from the initial radiographic images. We found that the initial deformity of the proximal humerus fracture in the coronal plane did not significantly affect the postoperative joint mobility and function; the varus, normal, and valgus groups were similar in anterior flexion, external rotation, and ASES and CMS function scores (*P* > 0.05). However, the varus group had a 20.7% complication rate, which was significantly higher than the normal and valgus groups (12.7% and 12.9%, respectively) (*P* < 0.05). This may be due to the different definitions of complications and the elderly patients we included. We defined complications as postoperative AVN, screw cut‐out, loss of reduction, deep infection and malunion, etc., and did not incorporate postoperative shoulder stiffness. This is similar to the results of some previous studies. Little *et al*.^16^ reported no significant differences in mean CMS and DASH functional scores between locking plate‐treated varus and valgus groups. Capriccioso *et al*.^11^ reported that locking plates use for proximal humerus fractures showed similar postoperative function in the varus and valgus groups, albeit with a higher complication rate in the former. However, Solberg *et al*.^17^ reported that the mean CMS in the varus group was significantly lower than in the valgus group (63.3 *vs* 71.2, respectively; *P* < 0.01). Unlike these studies, we included cases managed by internal fixation with plates and nails and classified the initial deformity into three groups: varus, normal, and valgus in the coronal plane. Based on our findings and previously published data, we conclude that the surgical treatment of proximal humeral fractures with plates and nails can achieve satisfactory results, in either initial varus or valgus deformities.

### Surgical Complications

We found an overall complication rate of 14.5% (19/131). The complication rate was 20.7% (6/29) in the varus group, 12.7% (9/71) in the normal group, and 12.9% (4/31) in the valgus group, with the varus group showing a significantly higher complication rate compared to the other two groups (*P* < 0.05). Published literature has reported complication rates of 10%–36% after the treatment of proximal humerus fractures with locking plates and 12%–59% after intramedullary nails.^18^, ^19^ The complication rate found in our study is relatively low, probably because our cohort was relatively young, had generally good bone stock, and 2‐part proximal humerus fractures accounted for nearly 1/3 of the cases. Several studies have reported that cases with an initial varus displacement have a higher risk of complication after proximal humerus fracture plate treatment^9^, ^11^, ^17^ because of the likelihood of destruction of the medial hinge, especially in cases with poor underlying bone quality.^19^ This places the implant at a significant mechanical disadvantage, thereby increasing the varus torque and stress on the locking screw. A study by Solberg *et al*.^17^ reported that in varus displaced fractures, approximately 71% of cases exhibited varus malreduction and that varus deformities over 5° were associated with a progressive sinking of the humeral head. Thus, the varus group shows a higher rate of complications because of the difficulty in maintaining the reduction of the humeral head. Our findings are similar in that our varus group showed a higher complication rate, even though we attempted to use calcar screws intraoperatively in all cases. In contrast to that study, the most common complications in our investigation involved screw cut‐out, AVN, and tuberosity malunions/resorption. In conclusion, for initial varus deformity of proximal humerus fracture, especially the elderly, the comminution of the medial column, bony conditions should be fully evaluated to choose the appropriate fixation method, but under the same conditions, the nail has better ability to maintain reduction.

### Maintenance of Reduction

Maintenance of reduction until bony union with resultant good shoulder function is the primary goal of surgical treatment of proximal humeral fractures. In the present study, we demonstrated that initial varus displacement, osteoporosis, medial comminution, and inadequate medial support are independent risk factors for loss of reduction during surgery of proximal humeral fractures.^20^ A study by Agudelo *et al*.^12^ showed that fixation failure occurred mainly in cases of initial varus deformity; they noted that an initial HSA of <120° was associated with a threefold higher risk of fixation failure than an HSA of >120°. Our findings show that the intraoperative NSA was returned to normal (−135°) in the varus, normal, and valgus groups, and no loss of reduction was detected at the last follow‐up. However, the varus group showed the largest NSA change of the three groups and, the NSA change in the plate group was significantly larger than in the medullary nail group, especially among the varus cases. This is similar to the findings of Bu *et al*.^21^ which reflects the biomechanical advantage of the nail in resisting varus and rotational stresses. Although no losses of reduction were noted at the last follow‐up, the changes in NSA suggest better maintenance of reduction with the nail, especially for initial varus displaced proximal humeral fractures.

### Strengths and Limitations

That study included cases treated with plate and nail fixation and, when grouped by initial deformity, we found that proximal humerus fractures with initial coronal displacement show similar postoperative function, and nailing provided better reduction and maintenance than locking plates, especially in varus fractures.

Nevertheless, some limitations are worth highlighting. The main one is the retrospective nature of this study. The surgical approach to locking plate osteosynthesis is affected by the type of fracture and physician preference, including the use of the deltopectoral or delta‐split approaches, which were not specifically distinguished in this study and may introduce some bias. Although the procedures were performed by senior surgeons, there are expected differences in intraoperative techniques that may have an impact on postoperative functions and complications.

### Conclusions

We grouped the cases treated with plate and nail fixation by initial coronal displacement (varus, normal, and valgus) and found that the three groups had similar postoperative function but a higher complication rate of varus fractures. Whereas the nail provides better maintenance of reduction relative to the locking plate, especially in varus fractures.

## Author Contributions

Dai Fei designed this study. Zhang Qing, Li Yiping and Yang Jinsong collected and analyzed the data. Dai Fei wrote the article. Xiang Ming revised the article. All the authors read and approved the final manuscript.
